# Comparison of monocyte human leukocyte antigen-DR expression and stimulated tumor necrosis factor alpha production as outcome predictors in severe sepsis: a prospective observational study

**DOI:** 10.1186/s13054-016-1505-0

**Published:** 2016-10-20

**Authors:** Anne M. Drewry, Enyo A. Ablordeppey, Ellen T. Murray, Evan R. Beiter, Andrew H. Walton, Mark W. Hall, Richard S. Hotchkiss

**Affiliations:** 1 Department of Anesthesiology, Washington University School of Medicine, 660 S. Euclid, St. Louis, MO USA; 2 Departments of Anesthesiology and Emergency Medicine, Washington University School of Medicine, St. Louis, MO USA; 3 University of Missouri-Columbia School of Medicine, Columbia, MO USA; 4 Department of Pediatrics, Critical Care Medicine, Nationwide Children’s Hospital, The Ohio State University College of Medicine, Columbus, OH USA

**Keywords:** Sepsis, Immunosuppression, Monocytes, Mortality

## Abstract

**Background:**

Identifying patients in the immunosuppressive phase of sepsis is essential for development of immunomodulatory therapies. Little data exists comparing the ability of the two most well-studied markers of sepsis-induced immunosuppression, human leukocyte antigen (HLA)-DR expression and lipopolysaccharide (LPS)-induced tumor necrosis factor alpha (TNF-ɑ) production, to predict mortality and morbidity. The purpose of this study was to compare HLA-DR expression and LPS-induced TNF-ɑ production as predictors of 28-day mortality and acquisition of secondary infections in adult septic patients.

**Methods:**

A single-center, prospective observational study of 83 adult septic patients admitted to a medical or surgical intensive care unit. Blood samples were collected at three time points during the septic course (days 1–2, days 3–4, and days 6–8 after sepsis diagnosis) and assayed for HLA-DR expression and LPS-induced TNF-ɑ production. A repeated measures mixed model analysis was used to compare values of these immunological markers among survivors and non-survivors and among those who did and did not develop a secondary infection.

**Results:**

Twenty-five patients (30.1 %) died within 28 days of sepsis diagnosis. HLA-DR expression was significantly lower in non-survivors as compared to survivors on days 3–4 (*p* = 0.04) and days 6–8 (*p* = 0.002). The change in HLA-DR from days 1–2 to days 6–8 was also lower in non-survivors (*p* = 0.04). Median HLA-DR expression decreased from days 1–2 to days 3–4 in patients who developed secondary infections while it increased in those without secondary infections (*p* = 0.054). TNF-ɑ production did not differ between survivors and non-survivors or between patients who did and did not develop a secondary infection.

**Conclusions:**

Monocyte HLA-DR expression may be a more accurate predictor of mortality and acquisition of secondary infections than LPS-stimulated TNF-ɑ production in adult medical and surgical critically ill patients.

**Electronic supplementary material:**

The online version of this article (doi:10.1186/s13054-016-1505-0) contains supplementary material, which is available to authorized users.

## Background

Sepsis-induced immunosuppression is a term used to describe the immunosuppressive phenotype that develops in many patients with protracted sepsis. It is characterized by numerous abnormalities in the innate and adaptive immune systems including increased immune cell apoptosis, impaired phagocytosis, diminished antigen-presenting ability, and dysregulated cytokine production [1]. Septic patients with these abnormalities are less able to eliminate primary infections and are more susceptible to secondary nosocomial infections, including those caused by opportunistic organisms [2].

Greater appreciation for the role of immune dysfunction in sepsis mortality has led to clinical trials of immunomodulatory agents that stimulate the immune system. Early evidence from these trials has been encouraging. Granulocyte-macrophage colony-stimulating factor (GM-CSF), a cytokine that activates neutrophils and monocytes, has been shown to reverse features of sepsis-induced immunosuppression and improve clinical outcomes such as hospital length of stay and acquisition of nosocomial infections [3, 4]. Interferon gamma (IFN-ɣ), also a potent monocyte activator, decreased time to fungal clearance in a randomized trial of HIV patients with cryptococcal meningitis [5]. Other potential immunotherapy agents, such as anti-programmed cell death ligand 1 (PD-L1) antibody and interleukin 7 (IL-7), have shown benefit in animal models of sepsis and are currently being tested in clinical trials [6, 7].

Immune responses during sepsis can vary among patients and evolve over the course of the illness. Ideally, only patients with features of immunosuppression should be treated with immunostimulatory agents to avoid potential harm in patients with already robust pro-inflammatory responses. Multiple assays for markers of immunosuppressive mechanisms have been developed to identify these patients [8, 9]. Two of the most well-studied markers of immunosuppression are ex vivo lipopolysaccharide (LPS)-induced tumor necrosis factor alpha (TNF-ɑ) production, which is a functional test of monocytic immune capacity, and monocyte cell surface expression of human leukocyte antigen (HLA)-DR. Decreased levels of TNF-ɑ production and HLA-DR expression indicate monocyte dysfunction and have been associated with an increased risk of nosocomial infections and death in critically ill patients [10–14]. Each of these markers has previously been used in clinical trials to identify patients who are at higher risk for morbidity and mortality in sepsis and who therefore might benefit from immunomodulatory therapy [3, 4].

While several studies have independently evaluated these markers in sepsis survivors and non-survivors, little data exist directly comparing the ability of HLA-DR expression and LPS-induced TNF-ɑ production to predict morbidity or mortality in adult septic patients [15–18]. Thus, the aim of this study was to determine which of these tests is most predictive of 28-day mortality and of acquisition of secondary infections in critically ill adult patients with severe sepsis.

## Methods

### Study design

This was a prospective observational study and is reported in accordance with the Strengthening the Reporting of Observational Studies in Epidemiology (STROBE) guidelines [19]. It was approved by the institutional Human Research Protections Office. Informed consent was obtained from all individual participants or their legally authorized representatives.

### Study setting and population

This study was conducted in the medical and surgical intensive care units (ICUs) of a 1200-bed university-affiliated tertiary care center between August 1, 2014 and May 31, 2015. Adult patients admitted to the ICU with a new diagnosis of severe sepsis within 48 hours were screened for inclusion. Severe sepsis was defined according to consensus criteria [20]. Exclusion criteria included: history of immunological disease, treatment with immunosuppressive medications within the previous 3 months, treatment with therapeutic hypothermia, and chronic infection with hepatitis B or C virus. Patients transferred to the institution from other hospitals with a diagnosis of sepsis were also excluded because the exact time of sepsis diagnosis could not be confirmed. Detailed inclusion and exclusion criteria are listed in Additional file 1.

### Data collection and clinical outcomes

Baseline demographics included age, sex, source of sepsis, presence of co-morbidities, Acute Physiology and Chronic Health Evaluation (APACHE) II score, and Sequential Organ Failure Assessment (SOFA) score. Microbiology data were collected for each positive culture. The primary outcome was 28-day mortality. The secondary outcome was acquisition of secondary infections. Secondary infections were defined as infections diagnosed by the treating physicians between 3 and 30 days after the primary sepsis diagnosis that required a new course of antibiotics for at least 5 days. The site of the secondary infection was determined by the presence of positive culture data or documentation in the medical record by the treating physicians.

### Immunological data

Blood samples were collected during three time periods for each patient, days 1–2 (time point A), days 3–4 (time point B), and days 6–8 (time point C) after sepsis diagnosis. The first 24-hour time period following the sepsis diagnosis time was considered to be day 1; the next 24-hour period, day 2; etc. These samples were tested for monocyte HLA-DR expression and LPS-induced TNF-ɑ production by blinded research assistants. Once patients were discharged from the ICU, subsequent blood samples were not collected. Additionally, leukocyte counts from complete blood cell counts (CBCs) ordered by the treating physicians were recorded for the first 7 days following sepsis diagnosis.

### Quantification of monocyte HLA-DR

**Fig. 1:**
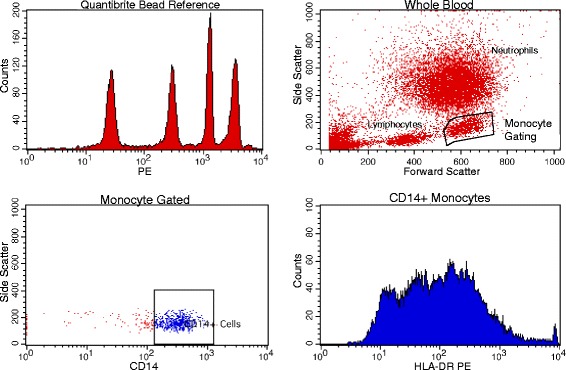
Gating strategy for determining monocyte HLA-DR levels. A monocyte gate was created by first gating upon forward and side scatter cell properties (*upper right panel*) and then further refining the monocyte gate by determining positivity for CD14 (*lower left panel*). Geometric mean fluorescence intensity (GMFI) data were then collected from the monocyte population in the HLA-DR channel (PE) (*lower right panel*) and compared against a Quantibrite Bead Reference (Becton Dickinson, San Jose, CA, USA) (*upper left panel*) to yield average per cell HLA-DR intensity. *HLA* human leukocyte antigen, *PE* phycoerythrin

Quantification of monocyte HLA-DR was performed according to the methods of Demaret et al. [21]. Briefly, whole blood was incubated with BD Quantibrite Anti-HLA-DR/Anti-Monocyte Stain (Becton Dickinson, San Jose, CA, USA), lysed using RBC Lysis Buffer (BioLegend, San Diego, CA, USA), and fixed in 2 % paraformaldehyde. Samples were acquired on a FACScan (Becton Dickinson, San Jose, CA, USA) with a five-color upgrade (CyTech, Fremont, CA, USA). Flow files were acquired and analyzed in CellQuest Pro (Becton Dickinson, San Jose, CA, USA). Antibodies bound per cell (ABC) were calculated by standardizing HLA-DR geometric mean fluorescence intensity (GMFI) of monocytes to BD Quantibrite-phycoerythrin (PE) beads (Becton Dickinson, San Jose, CA, USA) (Fig. 1).

### Quantification of LPS-induced TNF-ɑ production

LPS-stimulated whole blood TNF-ɑ production was performed according to the methods described by Hall et al. [4]. Patient blood was collected in sterile heparinized tubes and transferred to the laboratory within 5–15 minutes. Fifty microliters of whole blood was added to microcentrifuge tubes containing 0.5 mL RPMI-1640 fortified with 10 % fetal bovine serum, penicillin-streptomycin, non-essential amino acids, and either plus or minus 0.5 ng/mL LPS. The tubes were incubated at 37 °C for 4 hours. Supernatants were then removed and stored at -80 °C until enzyme-linked immunosorbent assays (ELISA) were performed for TNF-α production. All samples were run in duplicate. Additionally, a control population of five healthy volunteer adults was tested for TNF-α production.

### Statistical analysis

Descriptive statistics, including mean and standard deviation (SD) for normally distributed data or median and interquartile range (IQR) for non-normally distributed data, were used to describe the patient cohort. Normality was assessed using histograms and the Kolmogorov-Smirnov test. Comparisons of baseline characteristics in 28-day survivors and non-survivors were assessed using independent samples *t* tests, Mann-Whitney *U* tests, chi-square tests, or Fisher’s exact tests, as appropriate.

Analyses of HLA-DR expression and TNF-ɑ production were based on a repeated measures mixed model analysis. The primary focus of these analyses was on the significance of interactions that tested hypotheses regarding equality of changes over time in 28-day survivors and non-survivors and in those with and without a secondary infection. The appropriate statistical contrasts were used to test the null hypotheses that: (a) values at time points A, B, and C were equal between the groups; (b) change in values between time point A and time point B were equal between the groups; and (c) change in values between time point A and time point C were equal between the groups. These contrasts were not adjusted for multiple comparisons. Additionally, correlation between HLA-DR expression and LPS-induced TNF-ɑ production at each time point were evaluated with Pearson correlation after log transformation of the data.

## Results

**Fig. 2:**
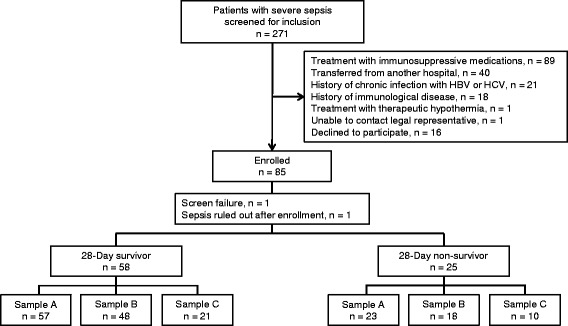
Flow diagram of included patients and reasons for patient exclusion. *HBV* hepatitis B virus, *HBC* hepatitis C virus

A total of 85 patients were enrolled after screening 271 patients with severe sepsis (Fig. 2). Two patients, one who was found to be a screen failure and another who was determined not to be infected following enrollment, were subsequently excluded from analysis.

Twenty-five patients (30.1 %) died within 28 days of sepsis diagnosis. Table 1 reports the baseline characteristics for survivors and non-survivors. Non-survivors had more severe disease than survivors as measured by APACHE II score (mean 22.6 [SD 5.8] vs 18.2 [5.0], *p* < 0.001) and SOFA score (mean 8.6 [SD 3.2] vs 6.9 [2.4], *p* = 0.009). There were no significant differences between the two groups in terms of age, gender, source of sepsis, proportion of patients with positive cultures, or type of infecting organism. Details regarding timing of blood sampling and reasons for missing samples in survivors and non-survivors are shown in Additional file 2. The median time from sepsis diagnosis until blood collection was not significantly different between the groups at any time point. Leukocyte counts in each group for the first 7 days after sepsis diagnosis are shown in Additional file 3.

**Table 1:** Baseline characteristics of 28-day survivors and non-survivors

	Survivors *n* = 58	Non-survivors *n* = 25	*p*
Age (years), mean (SD)	60.3 (17.5)	63.2 (17.8)	0.50
Sex (male), n (%)	38 (65.5)	13 (52.0)	0.24
APACHE II^a^, mean (SD)	18.2 (5.0)	22.6 (5.8)	<0.001
SOFA score^a^, mean (SD)	6.9 (2.4)	8.6 (3.2)	0.009
ICU type, n (%)			0.72
Medical	30 (51.7)	14 (56.0)	
Surgical	28 (48.3)	11 (44.0)	
Source of infection, n (%)			0.94
Lung	22 (37.9)	9 (36.0)	
Abdomen	11 (19.0)	5 (20.0)	
Urinary tract	8 (13.8)	5 (20.0)	
Bone or soft tissue	7 (12.1)	1 (4.0)	
Central line	3 (5.2)	1 (4.0)	
Endocarditis	2 (3.5)	1 (4.0)	
Undetermined	5 (8.6)	3 (12.0)	
Culture positive, n (%)	35 (60.3)	14 (56.0)	0.71
Organism, n (%)			0.88
Gram-negative	14 (24.1)	7 (28.0)	
Gram-positive	10 (17.2)	3 (12.0)	
Mixed	7 (12.1)	2 (8.0)	
Fungal	1 (1.7)	1 (4.0)	
Viral	3 (5.2)	1 (4.0)	
Co-morbidities, n (%)			
Coronary artery disease	15 (25.9)	6 (24.0)	0.55
Cerebrovascular disease	9 (15.5)	1 (4.0)	0.13
Congestive heart failure	16 (27.6)	8 (32.0)	0.79
Diabetes	21 (36.2)	3 (12.0)	0.034
Chronic renal insufficiency	14 (24.1)	4 (16.0)	0.56
Liver disease	4 (6.9)	3 (12.0)	0.42
COPD	14 (24.1)	2 (8.0)	0.13

*SD* standard deviation, *APACHE* Acute Physiology and Chronic Health Evaluation, *SOFA* Sequential Organ Failure Assessment, *ICU* intensive care unit, *COPD* chronic obstructive pulmonary disease

^a^Excluding neurological component

**Fig. 3:**
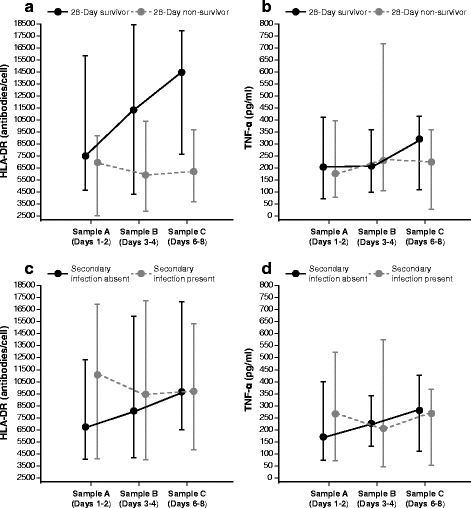
Median and interquartile range of (**a**) HLA-DR expression in survivors and non-survivors, (**b**) LPS-induced TNF-ɑ production in survivors and non-survivors, (**c**) HLA-DR expression in those who did and did not develop secondary infections, and (**d**) LPS-induced TNF-ɑ production in those who did and did not develop secondary infections. *HLA* human leukocyte antigen, *TNF-ɑ* tumor necrosis factor alpha

Figure 3a shows median and interquartile range of HLA-DR expression at each of the three time points (days 1–2 [A], days 3–4 [B], and days 6–8 [C]) in 28-day survivors and non-survivors. Table 2 shows results of the mixed models analysis comparing median HLA-DR expression at each time point and the median change in HLA-DR expression from time point A to time point B and from time point A to time point C between survivors and non-survivors. Survivors had significantly higher expression of HLA-DR at time point B (median 11,351 [IQR 4316, 18,417] antibodies (AB)/cell vs 5895 [2900, 10,388] AB/cell, *p* = 0.04) and at time point C (median 14,470 [7656, 17,906] AB/cell vs 6195 [3721, 9662] AB/cell, *p* = 0.002). Likewise, compared to non-survivors, survivors had a significantly greater increase in HLA-DR expression between time points A and C (*p* = 0.04). The change in HLA-DR expression between time points A and B was not statistically significant (*p* = 0.34). Septic patients had significantly lower TNF-ɑ production compared to healthy controls (median 195 [IQR 79, 373] pg/ml vs median 595 [IQR 500, 1302] pg/ml, *p* = 0.004). There were no significant differences in TNF-ɑ production at any time point between survivors and non-survivors (Fig. 3b and Table 2). The change in median TNF-ɑ production from time point A to B and from time point A to C was also not significantly different between the two groups (Table 2).

**Table 2:** Mixed models analysis of HLA-DR expression and LPS-induced TNF-ɑ production in 28-day survivors and non-survivors

		Survivors, *n* = 58	Non-survivors, *n* = 25	*p*
HLA-DR expression (antibodies/cell), median (IQR)	Sample A (days 1–2)^a^	7495 (4672, 15,824) *n* = 51	6971 (2525, 9162) *n* = 22	0.14
	Sample B (days 3–4)^a^	11351 (4316, 18,417) *n* = 43	5895 (2900, 10,388) *n* = 16	0.04
	Sample C (days 6–8)^a^	14470 (7656, 17,906) *n* = 18	6195 (3721, 9662) *n* = 10	0.002
	Δ Sample A to B	434 (-1710, 2921)	-630 (-1795, 626)	0.34
	Δ Sample A to C	1662 (-2645, 8118)	810 (-3382, 3507)	0.04
LPS-induced TNF-ɑ production (pg/ml), median (IQR)	Sample A (days 1–2)^a^	204 (73, 411) *n* = 50	176 (80, 396) *n* = 22	0.87
	Sample B (days 3–4)^a^	209 (101, 358) *n* = 40	232 (106, 716) *n* = 16	0.56
	Sample C (days 6–8)^a^	320 (111, 414) *n* = 17	225 (29, 358) *n* = 10	0.52
	Δ Sample A to B	0 (-130, 54)	20 (-23, 89)	0.27
	Δ Sample A to C	16 (-112, 85)	29 (-85, 141)	0.56

*HLA-DR* human leukocyte antigen-DR, *LPS* lipopolysaccharide, *TNF-ɑ* tumor necrosis factor alpha, *IQR* 25 %, 75 % interquartile range

^a^Days after sepsis diagnosis

Twenty-two patients (26.5 %) developed a secondary infection. The most common site of secondary infection was the lung (nine patients), and the most common cultured organisms were Gram-negative bacteria (Additional file 4). The median time to secondary infection onset was 9.4 days (IQR 5.5, 19.0 days). Table 3 shows the mixed models analysis for HLA-DR expression and LPS-induced TNF-ɑ production in patients who acquired secondary infections versus those who did not. There were no significant differences in HLA-DR values between these two groups at any of the three time points. However, patients who developed secondary infections demonstrated an overall decrease in HLA-DR expression from time point A to B (-934 [-6505, 353] AB/cell) while those who did not experienced an increase in HLA-DR expression 918 ([-1242, 4132] AB/cell, *p* = 0.54) (Fig. 3c and Table 3). There were no differences in TNF-α production at any time point nor changes in TNF-α production between time points A and B or between time points A and C (Fig. 3d and Table 3).

**Table 3:** Mixed models analysis of HLA-DR expression and LPS-induced TNF-ɑ production in septic patients who did and did not develop a secondary infection

		Secondary infection absent, *n* = 61	Secondary infection present, *n* = 22	*p*
HLA-DR expression (antibodies/cell), median (IQR)	Sample A (days 1–2)^a^	6761 (4054, 12,326) *n* = 53	11,133 (4054, 16,897) *n* = 20	0.38
	Sample B (days 3–4)^a^	8085 (4171, 15,951) *n* = 39	9438 (4008, 17,236) *n* = 20	0.79
	Sample C (days 6–8)^a^	9662 (6488, 17,165) *n* = 15	9722 (4852, 15,324) *n* = 13	0.77
	Δ Sample A to B	918 (-1242, 4132)	-934 (-6505, 353)	0.054
	Δ Sample A to C	3621 (-410, 9284)	-1479 (-3382, 1662)	0.32
LPS-induced TNF-ɑ production (pg/ml), median (IQR)	Sample A (days 1–2)^a^	171 (76, 400) *n* = 53	266 (73, 523) *n* = 19	0.42
	Sample B (days 3–4)^a^	227 (132, 341) *n* = 39	206 (46, 574) *n* = 17	0.27
	Sample C (days 6–8)^a^	282 (111, 427) *n* = 17	268 (52, 368) *n* = 10	0.91
	Δ Sample A to B	-3 (-137, 55)	16 (-48, 65)	0.61
	Δ Sample A to C	7 (-140, 97)	38 (-43, 120)	0.54

*HLA-DR* human leukocyte antigen-DR, *LPS* lipopolysaccharide, *TNF-ɑ* tumor necrosis factor alpha, *IQR* 25 %, 75 % interquartile range

^a^Days after sepsis diagnosis

**Fig. 4:**
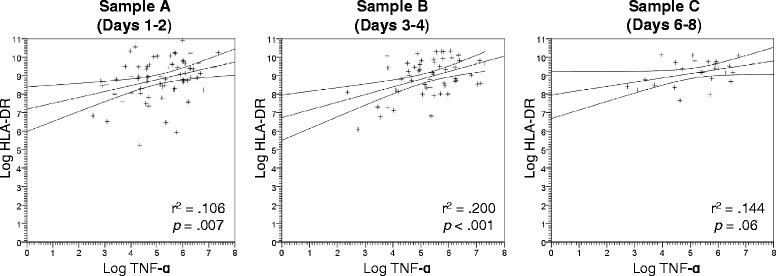
Plot of correlation between log-transformed HLA-DR expression and LPS-induced TNF-ɑ production for each measured time point (days 1–2, days 3–4, and days 6–8). *HLA* human leukocyte antigen, *TNF-ɑ* tumor necrosis factor alpha

Pearson correlation of log-transformed HLA-DR expression and LPS-induced TNF-ɑ production at time point A was 0.33 (*p* = 0.007); B, 0.45 (*p* < 0.001); and C, 0.38 (*p* = 0.06) (Fig. 4).

## Discussion

Investigators are employing a variety of methods to immune-phenotype septic patients. Numerous studies have shown that low monocyte HLA-DR expression identifies septic patients who are at higher risk for death or secondary hospital-acquired infections [10, 11, 22, 23]. Although not as extensively studied as monocyte HLA-DR expression, LPS-stimulated whole blood TNF-α production has also been used to identify immunosuppressed septic patients. Compared to monocyte HLA-DR expression, stimulated whole blood TNF-α production has a theoretical advantage as an indicator of host immunity because it is a reflection of actual cell function, i.e., the ability of cells to produce a key cytokine involved in host defense. Critically ill pediatric patients with persistently low stimulated TNF-α production have been shown to be more likely to acquire life-threatening infections, and importantly, treatment with immune-adjuvant GM-CSF was shown to cause a rapid improvement in stimulated TNF-α production that was associated with prevention of nosocomial infections [4, 14].

The purpose of the present study was to compare two tests that are used to evaluate immune status in septic patients. Results showed that quantification of monocyte HLA-DR expression could discriminate between sepsis survivors and non-survivors 3–4 days after sepsis onset, but not at days 1–2. These results are consistent with Monneret and associates who reported that only after 48 hours of sepsis duration did monocyte HLA-DR expression become predictive of survival [10]. We speculate that monocyte HLA-DR expression may not be predictive at an early phase of sepsis because circulating monocytes are likely recruited out of the bloodstream to sites of active infection. After this initial phase, a steady state phase may occur in which circulating monocytes more accurately reflect the whole body state of immunity. Results from this study also indicate that prediction of nosocomial infections may be best achieved by assessing changes in HLA-DR expression over time rather than values at individual time points.

One potential explanation for the usefulness of monocyte HLA-DR expression as a marker of immune status is its dynamic nature. Cell surface expression of HLA-DR is rapidly responsive to circulating levels of pro- and anti-inflammatory cytokines, which are essential in modulating the host response in sepsis. IL-6 and IL-10 cause downregulation of monocyte HLA-DR expression while, conversely, IL-12 and IFN-γ cause increased HLA-DR expression [24–26]. Also, HLA-DR plays a key role in T cell activation. Decreased HLA-DR expression may therefore result in less robust T cell stimulation and resultant reduced T cell cytokine production, proliferation, and cytotoxicity.

Although LPS-induced TNF-α production and HLA-DR expression were statistically correlated with each other, LPS-induced TNF-ɑ production was not significantly associated with mortality or acquisition of nosocomial infections at any measured time point. The lack of association between early LPS-induced TNF-α production and subsequent nosocomial infections is consistent with a previous study performed in critically ill adults [27]. The present study also showed that the TNF-α release assay conducted at later time points failed to predict clinical outcomes. There are several possible reasons for these results. The median value for TNF-α in septic patients was less than 250 pg/ml at every measured time point (except days 6–8 in the survivors), and the median value in healthy controls was lower than has been reported in previous studies [13, 18]. The narrower response range to LPS may have affected the ability to discriminate between survivors and non-survivors. Also, LPS-induced TNF-α production has primarily been studied in pediatric patients. There is increasing recognition of the impact of immunosenescence to blunt host response to infection [28]. We speculate that the increased age and high incidence of co-morbidities in this study contributed to a blunted TNF-α response in both survivors and non-survivors. TNF-α is largely produced by monocytes, so absolute numbers may affect overall TNF-α production. However, in the current study, survivors tended to have lower absolute monocyte counts than non-survivors, so this would not explain the low TNF-α values seen in survivors.

Another potential explanation for the superiority of HLA-DR expression over LPS-induced TNF-ɑ production as a predictor of poor outcomes in the present study is the differing degree to which these assays have been standardized. Monocyte HLA-DR quantitation was historically performed using flow cytometric methodology in which percent positivity was determined in a subject’s sample by comparison with a “negative” isotype control. Variability in cytometer settings and lot-to-lot variability in fluorochrome-labeled antibodies led to concern that the threshold of 30 % HLA-DR positivity as a definition of severe immunosuppression may not be generalizable between cytometers and institutions. The Quantibrite method employed in this study is also a flow cytometric test, but in this case, the number of molecules of HLA-DR per monocyte is calculated by comparing HLA-DR fluorescence in an antibody-labeled sampled with a set of standard beads with known HLA-DR expression. The use of standard beads reduces variability and has been shown to yield highly reproducible results across cytometers and institutions [29]. This is further evidenced by the fact that the HLA-DR values obtained in the present study are comparable to those previously published for similar septic cohorts [3, 30]. In contrast, LPS-induced TNF-ɑ production assays are more difficult to standardize. Recent data shows that inter-laboratory methodological variation (e.g., sample handling prior to stimulation, LPS source, LPS concentration, incubation time, etc.) impairs reproducibility and interpretation of results [31]. Increased standardization of this assay may improve the predicative performance of this biomarker.

Another key difference between these two assays is that the flow cytometry HLA-DR expression assay is specific to monocytes whereas the TNF-α production assay is performed using whole blood and thus evaluates the capacity of various cells to produce TNF-α in response to LPS. Although monocytes account for the majority of TNF-α production, lymphocytes and polymorphonuclear can produce TNF-α and other cytokines in significant amounts [32]. This may lead to increased variability in the TNF-α production assay as compared to HLA-DR expression assay.

Ultimately, sepsis-induced immunosuppression may be best diagnosed by assessing a combination of various biomarkers and clinical factors. In a recent study, monocyte PD-L1 expression, another potential marker of immune function, was found to accurately discriminate between 28-day survivors and non-survivors of sepsis, and its prognostic value was increased when assessed in combination with traditional predictors of mortality such as the SOFA score and Simplified Acute Physiology Score (SAPS) II score [33].

This study has several limitations. As expected, severity of illness differed among survivors and non-survivors, so it is not possible to conclude that HLA-DR expression is an independent predictor of mortality based on these results. Nonetheless, our aim was to compare HLA-DR expression and stimulated TNF-ɑ production as markers of mortality, which was achieved using univariable models. Also, while we used a very similar approach to the measurement of TNF-α response in recent pediatric studies, it is possible that differences in reagent preparation and assay performance had an effect on our results. Another limitation was that the number of patients included in analysis of time point C (days 6–8 after sepsis diagnosis) was limited due to patient death or discharge from the ICU. These missing samples could potentially have biased our results. The consistent upward trend in HLA-DR expression in survivors from days 1–2 to days 6–8 as compared to a decrease seen in non-survivors, however, supports the conclusion that lower HLA-DR expression is predictive of mortality at this later time point. TNF-α production, not found to be statistically different among survivors and non-survivors in this study, trends toward lower values in non-survivors on days 6–8. Perhaps, with greater statistical power, a significant difference would have been observed at this time point. Finally, this study does not address the ability of either of these tests to predict which patients might respond best to immunotherapy. Although patients with higher risks of mortality and acquisition of secondary infections are optimal targets for immunostimulatory therapy, the specific immunological tests best suited to specific therapies will need to be tested in clinical trials.

## Conclusions

The results of this study demonstrate that measurement of monocyte HLA-DR expression was a more accurate predictor of mortality and acquisition of nosocomial infections than LPS-stimulated TNF-ɑ production in a population of adult medical and surgical critically ill septic patients. However, since functional testing represents the gold standard for evaluating immune function, it is vitally important to improve performance and standardization of these assays to fully evaluate their role in the diagnosis of sepsis-induced immunosuppression. Additional studies are needed to clarify which biomarkers, or combinations of biomarkers, are potentially most useful for identifying patients who might benefit from immunotherapy.

## Additional files

Additional file 1: Detailed inclusion and exclusion criteria. (PDF 112 kb)
Additional file 2: Blood sampling details. (PDF 195 kb)
Additional file 3: Leukocyte counts in survivors and non-survivors for the first 7 days following sepsis diagnosis. (PDF 97 kb)
Additional file 4: Characteristics of secondary infections. (PDF 10 kb)

## Abbreviations

ABC: antibodies bound per cell
APACHE: Acute Physiology and Chronic Health Evaluation
CBC: complete blood cell count
ELISA: enzyme-linked immunosorbent assays
GM-CSF: granulocyte-macrophage colony-stimulating factor
GMFI: geometric mean fluorescence intensity
HLA: human leukocyte antigen
ICU: intensive care unit
IFN-γ: interferon gamma
IL-7: interleukin -7
IQR: interquartile range
LPS: lipopolysaccharide
PD-L1: programmed cell death ligand 1
PE: phycoerythrin
SD: standard deviation
SOFA: Sequential Organ Failure Assessment
TNF-ɑ: tumor necrosis factor alpha

## References

[1] Hotchkiss RS, Monneret G, Payen D (2013). Immunosuppression in sepsis: a novel understanding of the disorder and a new therapeutic approach. Lancet Infect Dis.

[2] Landelle C, Lepape A, Francais A, Tognet E, Thizy H, Voirin N, Timsit JF, Monneret G, Vanhems P (2008). Nosocomial infection after septic shock among intensive care unit patients. Infect Control Hosp Epidemiol.

[3] Meisel C, Schefold JC, Pschowski R, Baumann T, Hetzger K, Gregor J, Weber-Carstens S, Hasper D, Keh D, Zuckermann H, Reinke P, Volk HD (2009). Granulocyte-macrophage colony-stimulating factor to reverse sepsis-associated immunosuppression: a double-blind, randomized, placebo-controlled multicenter trial. Am J Respir Crit Care Med.

[4] Hall MW, Knatz NL, Vetterly C, Tomarello S, Wewers MD, Volk HD, Carcillo JA (2011). Immunoparalysis and nosocomial infection in children with multiple organ dysfunction syndrome. Intensive Care Med.

[5] Jarvis JN, Meintjes G, Rebe K, Williams GN, Bicanic T, Williams A, Schutz C, Bekker LG, Wood R, Harrison TS (2012). Adjunctive interferon-gamma immunotherapy for the treatment of HIV-associated cryptococcal meningitis: a randomized controlled trial. AIDS.

[6] Squibb B-M (2000). A phase 1b/2a, randomized, double-blinded, placebo-controlled, multicenter study to evaluate the safety, tolerability, pharmacokinetics and pharmacodynamics of BMS-936559 in subjects with severe sepsis. ClinicalTrials.gov [Internet].

[7] Revimmune (2000). A multicenter, randomized, double-blinded, placebo-controlled study of two dosing frequencies of recombinant interleukin-7 (CYT107) treatment to restore absolute lymphocyte counts in sepsis patients. ClinicalTrials.gov [Internet].

[8] Hamers L, Kox M, Pickkers P (2015). Sepsis-induced immunoparalysis: mechanisms, markers, and treatment options. Minerva Anestesiol.

[9] Venet F, Lukaszewicz AC, Payen D, Hotchkiss R, Monneret G (2013). Monitoring the immune response in sepsis: a rational approach to administration of immunoadjuvant therapies. Curr Opin Immunol.

[10] Monneret G, Lepape A, Voirin N, Bohe J, Venet F, Debard AL, Thizy H, Bienvenu J, Gueyffier F, Vanhems P (2006). Persisting low monocyte human leukocyte antigen-DR expression predicts mortality in septic shock. Intensive Care Med.

[11] Landelle C, Lepape A, Voirin N, Tognet E, Venet F, Bohe J, Vanhems P, Monneret G (2010). Low monocyte human leukocyte antigen-DR is independently associated with nosocomial infections after septic shock. Intensive Care Med.

[12] Lukaszewicz AC, Grienay M, Resche-Rigon M, Pirracchio R, Faivre V, Boval B, Payen D (2009). Monocytic HLA-DR expression in intensive care patients: interest for prognosis and secondary infection prediction. Crit Care Med.

[13] Muszynski JA, Nofziger R, Greathouse K, Nateri J, Hanson-Huber L, Steele L, Nicol K, Groner JI, Besner GE, Raffel C, Geyer S, El-Assal O, Hall MW (2014). Innate immune function predicts the development of nosocomial infection in critically injured children. Shock.

[14] Hall MW, Geyer SM, Guo CY, Panoskaltsis-Mortari A, Jouvet P, Ferdinands J, Shay DK, Nateri J, Greathouse K, Sullivan R, Tran T, Keisling S, Randolph AG, Pediatric Acute Lung Injury and Sepsis Investigators (PALISI) Network, PSI Flu Study Investigators (2013). Innate immune function and mortality in critically ill children with influenza: a multicenter study. Crit Care Med.

[15] Flohe S, Lendemans S, Schade FU, Kreuzfelder E, Waydhas C (2004). Influence of surgical intervention in the immune response of severely injured patients. Intensive Care Med.

[16] Docke WD, Randow F, Syrbe U, Krausch D, Asadullah K, Reinke P, Volk HD, Kox W (1997). Monocyte deactivation in septic patients: restoration by IFN-gamma treatment. Nat Med.

[17] Piani A, Hossle JP, Birchler T, Siegrist CA, Heumann D, Davies G, Loeliger S, Seger R, Lauener RP (2000). Expression of MHC class II molecules contributes to lipopolysaccharide responsiveness. Eur J Immunol.

[18] Ploder M, Pelinka L, Schmuckenschlager C, Wessner B, Ankersmit HJ, Fuerst W, Redl H, Roth E, Spittler A (2006). Lipopolysaccharide-induced tumor necrosis factor alpha production and not monocyte human leukocyte antigen-DR expression is correlated with survival in septic trauma patients. Shock.

[19] von Elm E, Altman DG, Egger M, Pocock SJ, Gotzsche PC, Vandenbroucke JP, Strobe Initiative (2007). The Strengthening the Reporting of Observational Studies in Epidemiology (STROBE) statement: guidelines for reporting observational studies. Ann Intern Med.

[20] Levy MM, Fink MP, Marshall JC, Abraham E, Angus D, Cook D, Cohen J, Opal SM, Vincent JL, Ramsay G, International Sepsis Definitions C (2003). 2001 SCCM/ESICM/ACCP/ATS/SIS International Sepsis Definitions Conference. Intensive Care Med.

[21] Demaret J, Walencik A, Jacob MC, Timsit JF, Venet F, Lepape A, Monneret G (2013). Inter-laboratory assessment of flow cytometric monocyte HLA-DR expression in clinical samples. Cytometry B Clin Cytom.

[22] Wu JF, Ma J, Chen J, Ou-Yang B, Chen MY, Li LF, Liu YJ, Lin AH, Guan XD (2011). Changes of monocyte human leukocyte antigen-DR expression as a reliable predictor of mortality in severe sepsis. Crit Care.

[23] Caille V, Chiche JD, Nciri N, Berton C, Gibot S, Boval B, Payen D, Mira JP, Mebazaa A (2004). Histocompatibility leukocyte antigen-D related expression is specifically altered and predicts mortality in septic shock but not in other causes of shock. Shock.

[24] Ohno Y, Kitamura H, Takahashi N, Ohtake J, Kaneumi S, Sumida K, Homma S, Kawamura H, Minagawa N, Shibasaki S, Taketomi A (2016). IL-6 down-regulates HLA class II expression and IL-12 production of human dendritic cells to impair activation of antigen-specific CD4(+) T cells. Cancer Immunol Immunother.

[25] Xiu B, Lin Y, Grote DM, Ziesmer SC, Gustafson MP, Maas ML, Zhang Z, Dietz AB, Porrata LF, Novak AJ, Liang AB, Yang ZZ, Ansell SM (2015). IL-10 induces the development of immunosuppressive CD14(+)HLA-DR(low/-) monocytes in B-cell non-Hodgkin lymphoma. Blood Cancer J.

[26] Salgado FJ, Lojo J, Fernandez-Alonso CM, Vinuela J, Cordero OJ, Nogueira M (2002). Interleukin-dependent modulation of HLA-DR expression on CD4and CD8 activated T cells. Immunol Cell Biol.

[27] van Vught LA, Wiewel MA, Hoogendijk AJ, Scicluna BP, Belkasim-Bohoudi H, Horn J, Schultz MJ, van der Poll T (2015). Reduced responsiveness of blood leukocytes to lipopolysaccharide does not predict nosocomial infections in critically ill patients. Shock.

[28] De Gaudio AR, Rinaldi S, Chelazzi C, Borracci T (2009). Pathophysiology of sepsis in the elderly: clinical impact and therapeutic considerations. Curr Drug Targets.

[29] Docke WD, Hoflich C, Davis KA, Rottgers K, Meisel C, Kiefer P, Weber SU, Hedwig-Geissing M, Kreuzfelder E, Tschentscher P, Nebe T, Engel A, Monneret G, Spittler A, Schmolke K, Reinke P, Volk HD, Kunz D (2005). Monitoring temporary immunodepression by flow cytometric measurement of monocytic HLA-DR expression: a multicenter standardized study. Clin Chem.

[30] Cheron A, Floccard B, Allaouchiche B, Guignant C, Poitevin F, Malcus C, Crozon J, Faure A, Guillaume C, Marcotte G, Vulliez A, Monneuse O, Monneret G (2010). Lack of recovery in monocyte human leukocyte antigen-DR expression is independently associated with the development of sepsis after major trauma. Crit Care.

[31] Segre E, Fullerton JN (2016). Stimulated whole blood cytokine release as a biomarker of immunosuppression in the critically ill: the need for a standardized methodology. Shock.

[32] Dubravec DB, Spriggs DR, Mannick JA, Rodrick ML (1990). Circulating human peripheral blood granulocytes synthesize and secrete tumor necrosis factor alpha. Proc Natl Acad Sci U S A.

[33] Shao R, Fang Y, Yu H, Zhao L, Jiang Z, Li CS (2016). Monocyte programmed death ligand-1 expression after 3-4 days of sepsis is associated with risk stratification and mortality in septic patients: a prospective cohort study. Crit Care.

