# Supplementary material for: Comparison of monocyte human leukocyte antigen-DR expression and stimulated tumor necrosis factor alpha production as outcome predictors in severe sepsis: a prospective observational study
Source: Crit Care. 2016 Oct 20;20:334. doi: 10.1186/s13054-016-1505-0 (PMC5072304; doi:10.1186/s13054-016-1505-0)
Supplement: Additional file 1: — Detailed inclusion and exclusion criteria. (PDF 112 kb) [file 13054_2016_1505_MOESM1_ESM.pdf]

Additional File 1. Detailed inclusion and exclusion criteria

Inclusion Criteria:

Admission to surgical or medical intensive care unit within 48 hours prior to enrollment

Age $\geq$18 years

Diagnosis of severe sepsis, defined by:

> Presence of proven or suspected infection (as indicated by order for antibiotics, excluding antibiotics ordered for perioperative prophylaxis)
>
> AND
>
> At least two of the following criteria present within 24 hours prior to enrollment: heart rate > 90 beats/minute, respiratory rate > 20 breaths/minute, white blood cell count < 4 or > 12 cells/µL x 10^3, or body temperature < 36.0°C or > 38.0°C
>
> AND
>
> At least one of the following criteria present within 24 hours prior to enrollment: mean arterial pressure < 65 mmHg or systolic blood pressure < 90 mmHg, requirement for vasopressors, lactate above normal limits, PaO_2/FiO_2 < 250, creatinine > 2.0 mg/dl (in patients with normal baseline renal function), bilirubin > 2.0 mg/dl, platelet count < 100,000, or INR > 1.5 (in patients not being treated with warfarin)

Exclusion Criteria:

Previous diagnosis of hematological or immunological disease (e.g. lymphoma, leukemia, myelodysplastic syndrome, HIV)

History of chronic infection with hepatitis B or C virus

Treatment with chemotherapy within 3 months

Treatment with oral or intravenous corticosteroids within 3 months

Treatment with therapeutic hypothermia in the intensive care unit prior to enrollment

Transferred directly to the intensive care unit from another hospital
