# Supplementary material for: Comparison of monocyte human leukocyte antigen-DR expression and stimulated tumor necrosis factor alpha production as outcome predictors in severe sepsis: a prospective observational study
Source: Crit Care. 2016 Oct 20;20:334. doi: 10.1186/s13054-016-1505-0 (PMC5072304; doi:10.1186/s13054-016-1505-0)
Supplement: Additional file 2: — Blood sampling details. (PDF 195 kb) [file 13054_2016_1505_MOESM2_ESM.pdf]

**Additional File 2.** Blood sampling details

| | **28-Day survivors, n = 58** | | | **28-Day non-survivors, n =25** | | |
|---|---|---|---|---|---|---|
| | **Sample A** | **Sample B** | **Sample C** | **Sample A** | **Sample B** | **Sample C** |
| Samples collected, n | 57 | 48 | 21 | 23 | 18 | 10 |
| Days after sepsis diagnosis, median (IQR) | 1.41 (1.04, 1.66) | 2.61 (2.38, 3.16) | 6.69 (5.82, 7.23) | 1.30 (0.94, 1.60) | 2.64 (2.28, 3.65) | 6.26 (5.62, 7.37) |
| Reason for missing samples, n | | | | | | |
| Discharged from ICU | 0 | 10 | 37 | 0 | 2 | 5 |
| Death prior to collection | 0 | 0 | 0 | 2 | 5 | 10 |
| Unable to draw blood | 1 | 0 | 0 | 0 | 0 | 0 |
| HLA-DR expression tested, n (%) | 51 (87.9) | 43 (74.1) | 18 (31.0) | 23 (92.0)[a] | 17 (68.8)[a] | 10 (40.0) |
| TNF-α production tested, n (%) | 50 (86.2) | 40 (69.0) | 17 (29.3) | 23 (92.0)[a] | 17 (68.0)[a] | 10 (40.0) |

[a]Immunological data from one non-survivor was excluded from analysis due to being a statistical outlier. IQR, 25%, 75% interquartile range; ICU, intensive care unit; HLA-DR, human leukocyte antigen-DR; TNF-α, tumor necrosis factor-alpha
