# Supplementary material for: Comparison of monocyte human leukocyte antigen-DR expression and stimulated tumor necrosis factor alpha production as outcome predictors in severe sepsis: a prospective observational study
Source: Crit Care. 2016 Oct 20;20:334. doi: 10.1186/s13054-016-1505-0 (PMC5072304; doi:10.1186/s13054-016-1505-0)
Supplement: Additional file 3: — Leukocyte counts in survivors and non-survivors for the first 7 days following sepsis diagnosis. (PDF 97 kb) [file 13054_2016_1505_MOESM3_ESM.pdf]

Additional File 3. Leukocyte counts in survivors and non-survivors for the first seven days following sepsis diagnosis

	**Day 1**	**Day 2**	**Day 3**	**Day 4**	**Day 5**	**Day 6**	**Day 7**
				**28-Day Survivors**			
No. of samples	57	50	53	49	48	32	35
WBC, (cells/µL x 10^3), median (IQR)	11.9 (8.8, 17.5)	10.0 (6.5, 14.5)	8.2 (5.8, 13.5)	8.8 (6.4, 10.7)	9.5 (6.0, 12.8)	9.4 (5.6, 14.9)	8.7 (6.2, 12.3)
ANC, (cells/µL x 10^3), median (IQR)	9.6 (7.1, 15.4)	8.0 (5.2, 11.4)	6.9 (4.2, 10.2)	6.5 (4.2, 7.9)	6.7 (5.2, 9.9)	7.8 (4.1, 12.9)	7.1 (4.3, 10.5)
ALC, (cells/µL x 10^3), median (IQR)	1.0 (0.5, 1.6)	0.9 (0.6, 1.7)	1.0 (0.6, 1.4)	1.1 (0.8, 1.4)	1.2 (0.7, 1.7)	1.1 (0.7, 1.6)	1.2 (0.9, 1.5)
AMC, (cells/µL x 10^3), median (IQR)	0.6 (0.5, 1.0)	0.5 (0.4, 0.8)	0.6 (0.5, 0.9)	0.6 (0.4, 0.7)	0.6 (0.5, 0.9)	0.8 (0.5, 0.9)	0.6 (0.4, 0.9)
				**28-Day Non-Survivors**			
No. of samples	25	23	20	18	15	15	13
WBC, (cells/µL x 10^3), median (IQR)	14.3 (11.9, 21.1)	14.8 (8.6, 20.5)	14.0 (9.6, 19.3)	13.2 (9.5, 20.4)	13.9 (7.6, 23.3)	14.3 (10.0, 20.3)	15.9 (10.8, 21.0)
ANC, (cells/µL x 10^3), median (IQR)	12.9 (10.3, 20.8)	12.1 (6.2, 16.0)	10.9 (7.5, 16.9)	11.9 (7.4, 18.0)	11.5 (6.8, 19.8)	13.4 (8.6, 18.4)	15.6 (8.3, 19.6)
ALC, (cells/µL x 10^3), median (IQR)	1.0 (0.6, 1.4)	1.0 (0.5, 1.4)	1.0 (0.7, 1.5)	0.9 (0.7, 1.3)	1.1 (0.6, 1.4)	1.1 (0.5, 1.4)	1.2 (0.9, 1.4)
AMC, (cells/µL x 10^3), median (IQR)	0.9 (0.6, 1.2)	0.8 (0.3, 1.3)	0.9 (0.5, 1.3)	0.8 (0.7, 1.3)	0.6 (0.4, 1.1)	0.9 (0.7, 1.4)	0.9 (0.6, 1.6)

WBC, white blood cell; IQR, 25%, 75% interquartile range; ANC, absolute neutrophil count; ALC, absolute lymphocyte count; AMC, absolute monocyte count.
