# Supplementary material for: Comparison of monocyte human leukocyte antigen-DR expression and stimulated tumor necrosis factor alpha production as outcome predictors in severe sepsis: a prospective observational study
Source: Crit Care. 2016 Oct 20;20:334. doi: 10.1186/s13054-016-1505-0 (PMC5072304; doi:10.1186/s13054-016-1505-0)
Supplement: Additional file 4: — Characteristics of secondary infections. (PDF 10 kb) [file 13054_2016_1505_MOESM4_ESM.pdf]

Additional File 4. Characteristics of secondary infections

	**Patients with secondary infections, n = 22**
Time to secondary infection (days), median (IQR)	9.4 (5.5, 19.0)
Site of secondary infection, n (%)	
Lung	9 (40.9)
Abdomen	7 (31.8)
Urinary tract	1 (4.5)
Blood	1 (4.5)
Wound	1 (4.5)
Other/Unknown	3 (13.6)
Culture positive, n (%)	9 (40.9)
Organism, n (%)	
Gram-negative	4 (18.2)
Gram-positive	1 (4.5)
Mixed	1 (4.5)
Fungal	1 (4.5)
Viral	2 (9.1)

IQR, 25%, 75% interquartile range
